# An early phase of instructive plasticity before the typical onset of sensory experience

**DOI:** 10.1038/s41467-019-13872-1

**Published:** 2020-01-02

**Authors:** Arani Roy, Shen Wang, Benyamin Meschede-Krasa, Jordan Breffle, Stephen D. Van Hooser

**Affiliations:** 1 Department of Biology, Brandeis University, Waltham, MA 02454 USA; 2 Volen Center for Complex Systems, Brandeis University, Waltham, MA 02454 USA; 3 Sloan-Swartz Center for Theoretical Neurobiology, Brandeis University, Waltham, MA 02454 USA

**Keywords:** Neuronal development, Neural circuits

## Abstract

While early experience with moving stimuli is necessary for the development of direction selectivity in visual cortex of carnivores, it is unclear whether experience exerts a permissive or instructive influence. To test if the specific parameters of the experienced stimuli could instructively sculpt the emergent responses, visually naive ferrets were exposed to several hours of experience with unusual spatiotemporal patterns. In the most immature ferrets, cortical neurons developed selectivity to these patterns, indicating an instructive influence. In animals that were 1–10 days more mature, exposure to the same patterns led to a developmentally-typical increase in direction selectivity. We conclude that visual development progresses via an early phase of instructive plasticity, when the specific patterns of neural activity shape the specific parameters of the emerging response properties, followed by a late phase of permissive maturation, when sensory-driven activity merely serves to enhance the response properties already seeded in cortical circuits.

## Abstract (web summary)

Brain circuits exhibit different amounts of plasticity over different developmental stages. Here authors show that there is a transition of the influence of spatiotemporal patterns, from instructive to permissive, around the time of the onset of visual experience.

## Introduction

In the developing visual system, molecular cues^1,2^, and early spontaneous activity^3–7^ lay down the foundation of initial circuitry that exhibits many of the properties that are found in the mature animal, including retinotopic organization and orientation selectivity^8–12^. During a subsequent phase of experience-dependent development, visually-driven activity further shapes these response properties, providing enhanced cortical acuity^13^, binocular matching of inputs from the 2 eyes^14^, and, in carnivores and primates, the emergence of direction-of-motion selectivity^15,16^. It is of particular interest to understand how early visual activity interacts with, and alters, the immature circuit. Do the circuit connections established before the onset of experience commit cortex to a developmental path with pre-destined response properties, such that subsequent sensory experience merely permits maturation of these pre-seeded properties? Or is the cortical circuit malleable enough so that the particular patterns of visually-driven activity can instructively sculpt the responses according to the quality of the specific stimuli experienced?

Direction selectivity—a preference for stimulus movement in 1 direction as opposed to all others—typically develops in ferret visual cortex over a period of 1–2 weeks after eye-opening through a process that requires visual experience^15,17,18^, and does not form in dark-reared^15^ or strobe-reared^19–22^ animals. Direction selectivity can also be rapidly induced in the laboratory by providing an anesthetized ferret kit with 3–9 h of experience with drifting gratings^17,18,23,24^. While exposure to such smooth spatiotemporal motion increases direction selectivity, many parameters of direction tuning are invariant to the specific parameters of the gratings used for visual stimulation. For example, orientation selectivity is barely malleable during motion exposure: only columns whose orientation preference match the provided stimulus exhibit increases in direction selectivity^17^, and the orientation preferences of neurons that initially prefer other orientations are changed only very slightly^17^. Direction angle preference is also relatively unchangeable: stimulation with gratings that move in only one direction cause a dramatic increase in direction selectivity for cells whose initial biases match the stimulated direction, but do not cause an increase in selectivity for cells whose initial biases match the opposite direction^23^. Speed/temporal frequency tuning is also invariant: stimulation with either slow or fast moving stimuli causes an increase in direction selectivity, but does not alter tuning for speed/temporal frequency^24^. These results suggest that experience with drifting gratings fails to modify many of the parameters of direction tuning (orientation/direction preference angle, speed/temporal frequency, etc.), thereby implying that visual experience is only necessary to permissively increase selectivity and acuity of the tuning.

While the above results suggest a limit to the extent to which the experienced stimulus can shape cortical tuning properties, no experiment to date has directly tested if the nascent visual cortex can be induced to develop selective responses to irregular spatiotemporal patterns, which would be a strong test of whether selectivity is instructed by activity. In all visual motion stimulation experiments to date, young ferrets were exposed to smoothly moving gratings, in which an oriented grating is moved along a smoothly progressing sequence of spatial phases in time. According to the spatiotemporal receptive fields of neurons in the typically-developed visual cortex, such stimuli are ideally suited for driving cortical neurons^25–27^. In addition, the vast majority of ferret kits examined in prior studies already had visual experience for 1–3 days at the time of each experiment, making it difficult to rigorously assess if activity before or around the time of natural eye-opening could instructively modify the cortex.

To address these issues, we directly manipulated early visual experience by prematurely opening the eyes of young ferrets and exposing them to grating stimuli that were animated with scrambled spatiotemporal phase sequences. We reasoned that if the patterns of early activity in visual circuits were instructive, then we should be able to induce increased responses to these phase-scrambled grating stimuli through repeated visual exposure. On the other hand, if the cortical circuitry were already committed to developing selectivity for smooth motion, then providing phase-scrambled stimulation should merely increase direction selectivity.

We found evidence for a transition of the influence of early activity in the visual cortex—from instructive to permissive—that occurred around the time of natural eye-opening. When the eyes were opened prematurely, or if the state of the cortex was very immature as assessed by levels of orientation selectivity, animals developed increased selectivity to the artificial phase-scrambled stimulus that was experienced. Animals that were slightly more mature did not acquire increased selectivity to the phase-scrambled patterns but instead exhibited a developmentally-typical increase in direction selectivity, consistent with a permissive influence of visual stimulation. These data provide evidence that the early activity in visual cortex that occurs before and at eye-opening—which includes spontaneous activity^3–5^, low resolution visual stimulation through the closed lids^28,29^, and higher resolution vision through the slowly opening eyes—provides an instructive signal for neural circuit construction. Later activity, after the normal onset of visual experience, is necessary for the maturation of direction selectivity, but only in a permissive manner.

## Results

### Designing motion stimuli with irregular spatiotemporal phase

**Fig. 1:**
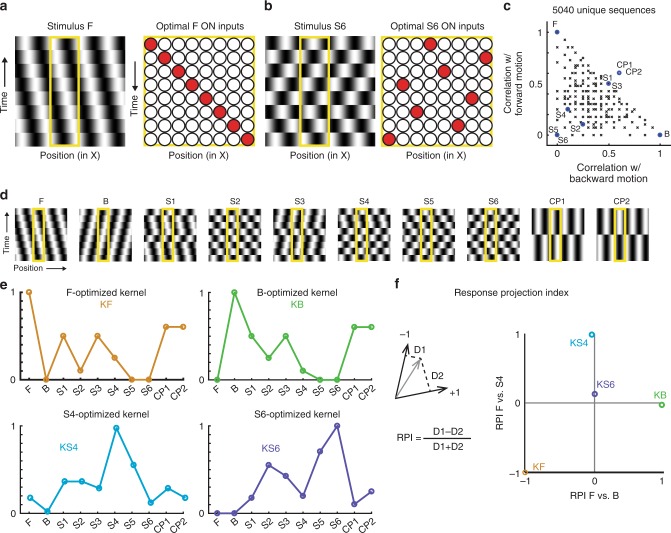
Design of a spatiotemporal stimulus family. **a** Left: X-T (space-time) view of vertical sinusoidal grating shifting to left at each step, termed forward stimulus (F). Each strip represents video frame. Yellow box indicates hypothetical receptive field. Right: Hypothetical X-T inputs (ON only) that depict positions, latencies of inputs that would drive optimal response to F. **b** Left: X-T view of vertical sinusoidal grating (stimulus S6) with scrambled phase steps [8 3 6 2 7 4 1 5]. Right: Hypothetical X-T inputs (ON only) that depict positions, latencies of inputs that would drive optimal response to S6. **c** Plot of best-aligned correlation with forward (F), backward (B) motion for all 5040 unique phase sequences (black x) and our selections for stimuli (blue circles). F, B represent forward and backward smooth motion; S1–S6 are phase-scrambled stimuli that deviate substantially from F, B; CP1, CP2 are counterphase stimuli. **d** Video frame strips of all stimuli. **e** Responses of hypothetical cells with input kernels optimized for indicated stimuli. Cell optimized for F (KF, orange) responds strongly to F, but not to B, S5, or S6. Cell optimized for B (KB, green) responds strongly to B but not F, S5, or S6. Cells optimized for either S4 (KS4, cyan) or S6 (KS6, purple) respond weakly to F and B and poorly to each other’s optimal stimulus. **f** Response Projection Index (RPI) indicates how tuning curve of a given cell matches those of cells optimized for 2 stimuli. Left: Each cell’s normalized response curve in 10-dimensional space. Response is compared to the responses expected from hypothetical reference neurons optimized for 2 stimuli (such as F and B). Distance in vector space between actual response (gray vector) and vector line defined by reference neurons is calculated (D1 and D2), and index is calculated RPI = (D1 − D2)/(D1 + D2). If cell’s responses match that of hypothetical neuron optimized for first (second) reference stimulus, then RPI is −1 (1). Right: Scatter plot of RPI index values for kernels optimized for stimuli indicated. X-axis is RPI relative to F, B and Y-axis is RPI relative to F, S4.

Neurons in carnivore primary visual cortex respond strongly to oriented gratings moving in one direction following a smoothly progressing sequence of spatiotemporal phases. We wanted to test if early exposure to gratings moving with irregular spatiotemporal patterns could modify the cortical circuitry and induce neurons to respond selectively to irregular motion. For this purpose, we designed a stimulus family of gratings moving with scrambled spatiotemporal phase sequences. To create such phase-scrambled visual stimuli, we varied the typical oriented grating stimuli that drive the cortex well. We discretized grating phase into 8 steps (Fig. 1), defined forward (F) and backward (B) stimuli as phase sequences [1 2 3 4 5 6 7 8] and [8 7 6 5 4 3 2 1], respectively, and approximated a viewing temporal frequency of 2 Hz by showing each phase for 1/(8*2 Hz) = 0.0625 s. We quantitatively analyzed the set of possible 5040 unique sequences (see “Methods” section; Fig. 1c, Supplementary Figs. 1, 2), and evaluated their degree of similarity to smooth motion. Subsequently, we chose for experiments a family of 10 sequences, containing a mixture of low and intermediate levels of similarity to smooth motion: forward motion (F), backward motion (B), 6 sequences that exhibited varying degrees of correlation with forward and backward motion (scrambled: S1–S6), and counterphase stimuli at 2 spatial phases labeled CP1 and CP2, respectively (Fig. 1a–d**;** Supplementary Movies 1–10). Stimuli S1–S6 contain spatiotemporal energy at multiple spatial and temporal frequencies (Supplementary Fig. 2), while stimuli F, B, CP1, and CP2 contain energy around a single spatial and temporal frequency. S4 and S6 were chosen for visual stimulation due to their very low correlation with smooth motion, while all 10 sequences were used to test responses before and after visual stimulation.

We developed 2 selectivity measures to quantify neural responses to this stimulus family—the response set for each neuron being 10-dimensional due to the inclusion of 10 stimuli in the experiment. The first measure, called the selectivity index (SI) for stimulus *n*, is equal to the response of the neuron to that stimulus divided by the sum of the responses to the chosen 10 stimuli. We also developed a second measure called the response projection index (RPI), which considered the fact that F…CP2 are correlated with one another to varying degrees. We can imagine linear receptive field kernels (*KX*) that would give a maximal response to a stimulus (*X*), as shown in Fig. 1e, and we can compute the responses of these kernels to each of the 10 stimuli. The RPI describes how close the response of a measured neuron, in 10-dimensional response space, is to the responses that would be expected from an ideal kernel (*KX*) relative to another ideal kernel (*KY)* (Fig. 1f). A neuron that gives responses identical to KF would have an RPI(KF vs KB) value of −1, whereas a neuron that gives responses that are identical to KB would have an RPI(KF vs KB) value of +1. Stimulus S6 is uncorrelated with F and B, and KS6 has an RPI(KF vs KB) value of 0 (Fig. 1f).

We prepared young ferrets for 2-photon imaging of virally-expressed GCaMP6s in visual cortex^30^ (see “Methods” section). We began each experiment by measuring responses to drifting gratings in order to assess the initial orientation and direction tuning. A well-represented orientation preference was selected to be the orientation angle for stimuli [F…CP2], and responses to these stimuli were measured. Initial responses were measured at a single depth in order to limit stimulation that could alter the receptive fields. Next, stimulus S4 or S6 (stimuli that were poorly correlated with F and B) was selected for 6 h of prolonged visual stimulation^17,23,24^; in previous studies, 3–6 h of visual stimulation was sufficient to drive substantial increases in direction selectivity, and animals remained physiologically robust over this time. Finally, responses to stimuli [F…CP2] and traditional orientation and direction tuning were re-assessed using sinusoidal gratings. In a few experiments, we were able to track some of the same cells over time, but in most experiments cells expressing gCamp6s were dark when unstimulated, and the exact alignment of imaging fields was not performed.

### Exposure to irregular motion: individual examples

**Fig. 2:**
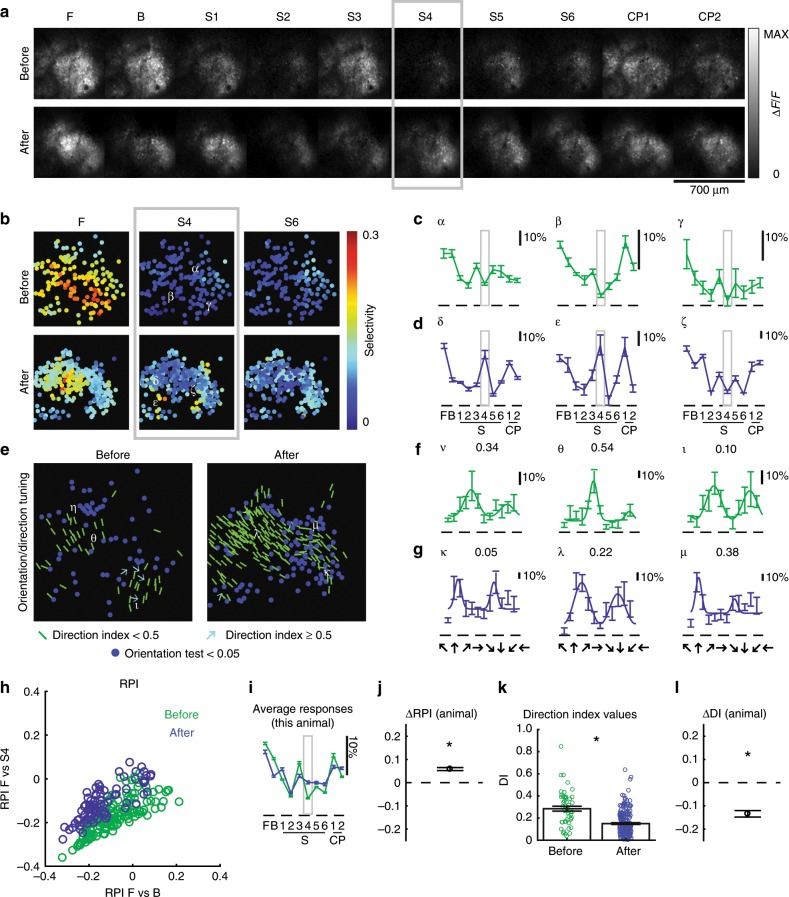
In naive ferret, 6 h experience with phase-scrambled grating pattern caused an increase in pattern selectivity. **a** GCaMP6s responses to the family of spatiotemporal stimuli before, after 6 h of training with pattern S4, indicating substantial increase in selectivity for S4. Animal’s eyes were opened prematurely on P30. **b** Single cell selectivity index (SI) values for different stimuli (F—a phase-regular, unscrambled direction stimulus, and S4/S6—phase-scrambled stimuli). Selectivity for F decreases, while selectivity for S4 increases.  All visually-responsive cells included. **c** Responses to example cells (in B) before, **d** after experience. Error bars are standard error of mean (SEM) across trials. Cells δ, ε exhibit strong responses to S4. **e** Orientation, direction tuning before, after training. Blue dots represent visually-responsive cells that do not exhibit significant variation across direction stimuli; green bars represent orientation-selective but not strongly direction-selective cells (DI < 0.5), cyan arrows indicate strongly direction-selective cells (DI ≥ 0.5). **f** Direction tuning of example cells (in **e**) before, **g** after the experience. Numbers indicate direction index values. Error bars are SEM. **h** Response Projection Index (RPI) for F vs B (X-axis) and F vs S4 (Y-axis) for cells measured before (green) and after (blue) 6 h of experience with S4. There is an upward shift on Y-axis, indicating cells exhibit responses more like cell optimized to S4. **i** Grand average of responses before and after 6 h exposure to S4. On average, there is an enhancement of the response to S4. Error bars are SEM across cells. **j** Difference in cell RPI (F vs S4) before (*N* = 138 cells), after (*N* = 82 cells) experience (error bars: 95% confidence intervals), indicating significant increase in selectivity to S4. * indicates 95% confidence interval does not include 0. Cells that exhibited significant variation across scrambled stimuli included.  **k** Direction index values before (*N* = 50 cells), after (*N* = 200 cells) exposure to S4. Direction index values decreased slightly after exposure to S4. Error bars are SEM across cells. * Indicates 95% confidence interval does not include 0 (see **l**). Cells that exhibited significant variation across direction stimuli included. **l** Estimated difference in DI of cells before, after the experience (error bars are 95% confidence intervals), indicating significant decrease in DI with S4 experience. * Indicates 95% confidence interval does not include 0.

**Fig. 3:**
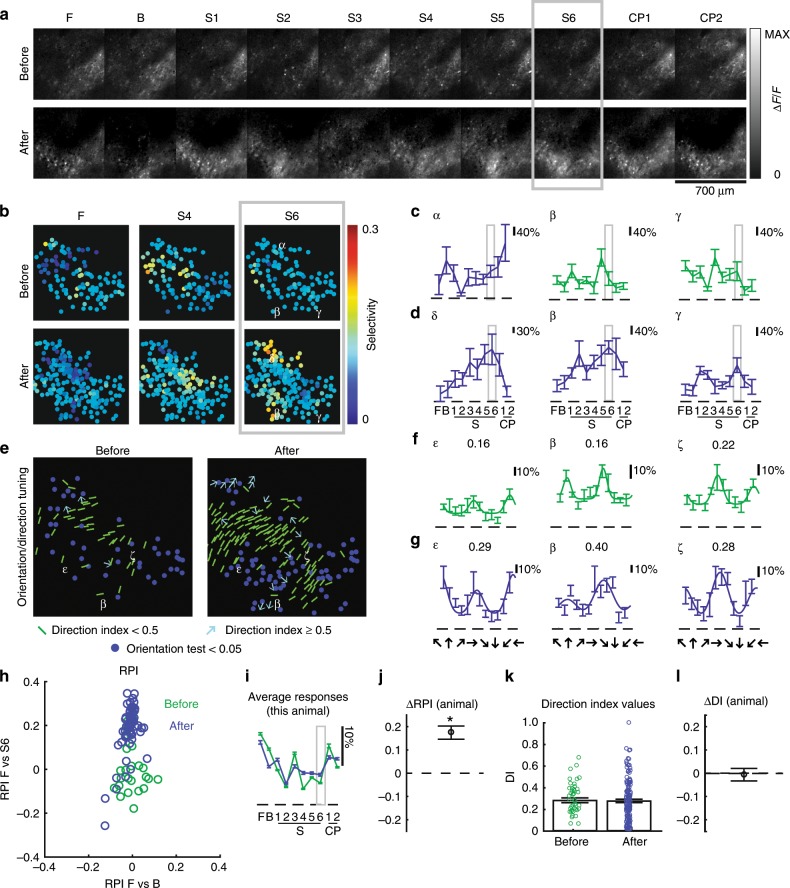
In a second visually naive ferret, 6 h of experience with a phase-scrambled grating pattern caused an increase in selectivity for that pattern. The eyes were opened prematurely on P31. Panels **a**–**l** are as described in Fig. 2, except that S6 was used as the training stimulus. In this animal, some cells were tracked across time and Greek letters appear more than once. This animal exhibited increased selectivity to training stimulus S6, and no significant alterations to direction selectivity. *N* = 25 cells before and *N* = 62 cells after for measurements of F, B, S1–6, CP1–2, and *N* = 41 cells before and *N* = 145 cells after for direction tuning. Premature animals varied in the influence of phase-scrambled grating patterns on traditional direction selectivity.

Example responses from a ferret (age P30) whose eyes were opened prematurely are plotted in Fig. 2. After 6 h of exposure to stimulus S4, there is a clear enhancement of the response of the imaging field to stimulus S4 (Fig. 2a–d, h–j). To characterize the degree to which neural responses were similar to that of a neuron that is perfectly selective to the trained stimulus S4, we computed the RPI for stimulus F vs B and F vs S4. There is a clear upward shift in RPI F vs S4, indicating that neural responses are more selective for stimulus S4 after exposure than before (Fig. 2h–j). Despite the fact that the ferret received stimulation with the relatively broadband motion stimulus S4, traditional direction selectivity index values for this animal exhibited a decrease (Fig. 2d–f, k, l), which is opposite to what we would have expected if the visual experience were only capable of inducing permissive changes^17,23,24^. Responses from another ferret (age P31) whose eyes were opened prematurely are shown in Fig. 3. This animal was shown stimulus S6 for 6 h, and also exhibited an increase in response to stimulus S6 (Fig. 3a–d, h–j). This animal exhibited no significant change in direction selectivity (Fig. 3d–f, k, l), indicating that selectivity was reconfigured in a manner that, while closer to a hypothetical neuron that would respond to stimulus S6, did not change significantly in terms of direction selectivity.

**Fig. 4:**
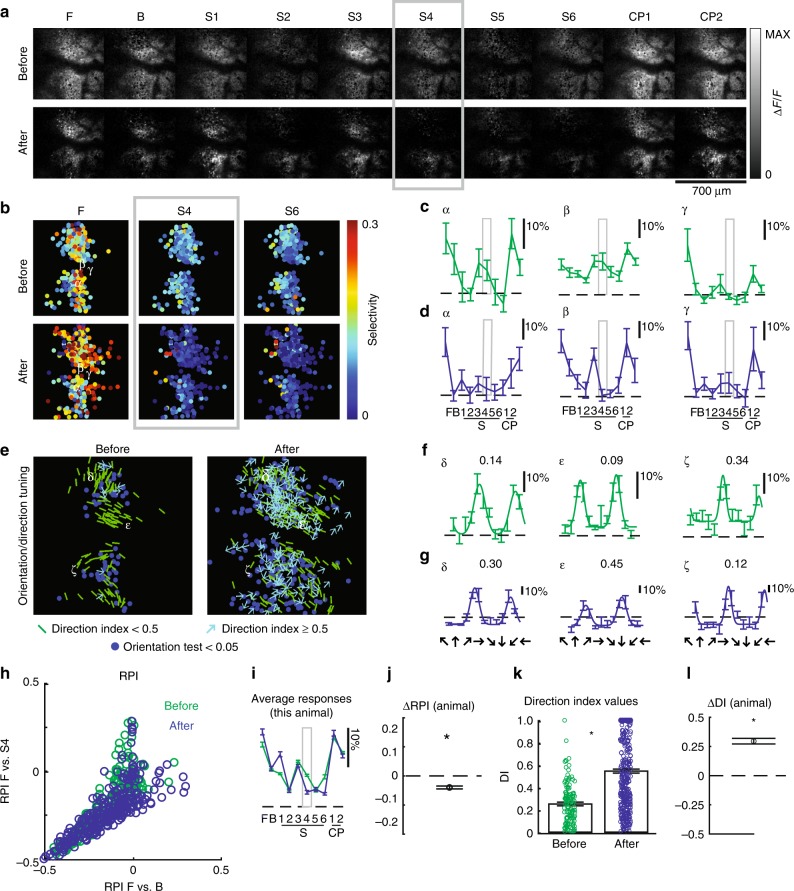
In a ferret with 3 days of visual experience, 6 h of experience with a phase-scrambled grating pattern caused an increase in direction selectivity rather than selectivity for the phase-scrambled pattern. The eyes opened naturally on day P33, and the experiment was performed at P36. Panels **a**–**l** are as described in Figs. 2, 3. S4 was used as the training stimulus. In this animal, some cells were tracked across time and Greek letters appear more than once. *N* = 170 cells before and *N* = 267 cells after for measurements of F, B, S1–6, CP1–2, and *N* = 124 cells before and *N* = 371 cells after for direction tuning. This animal exhibited decreased selectivity to training stimulus S4, and exhibited a significant increase in direction selectivity.

**Fig. 5:**
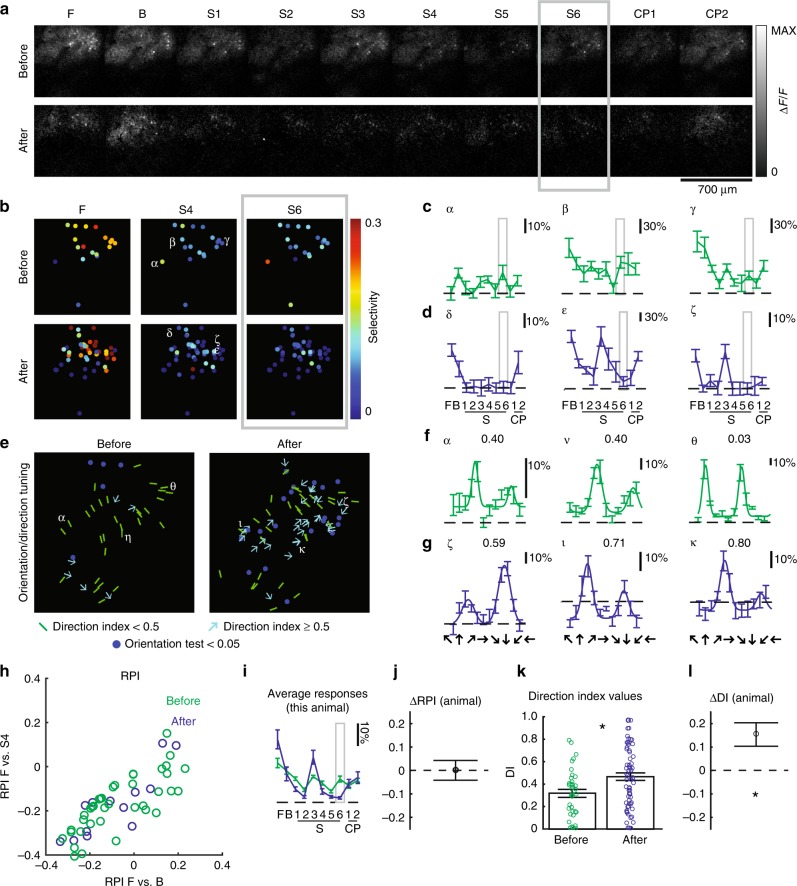
In a ferret with 5 days of visual experience, 6 h of experience with a phase-scrambled grating pattern caused an increase in direction selectivity rather than selectivity for the phase-scrambled pattern. The eyes opened naturally on day P33, and the experiment was performed at P38. Panels a–l are as described in Figs. 2–4. S6 was used as the training stimulus. In this animal, some cells were tracked across some trials and Greek letters appear more than once. *N* = 13 cells before and *N* = 41 cells after for measurements of F, B, S1–6, CP1–2, and *N* = 36 cells before and *N* = 67 cells after for direction tuning. This animal exhibited no change in selectivity to training stimulus S6, and exhibited a significant increase in direction selectivity.

Responses in slightly more mature ferrets resembled the example in Fig. 4. This ferret (age P36, 3 days of natural visual experience) was also exposed to stimulus S4 for 6 h, following which there was no increase in selectivity to stimulus S4 (Fig. 4a–d, h–j). As a result, there was no upward shift in RPI F vs S4, in fact there was a small but significant downward shift (Fig. 4h–j). Instead, this animal exhibited an increase in direction selectivity index values, (Fig. 4d–f, k, l). A second example from an older animal (age 38, 5 days of visual experience) is shown in Fig. 5. After 6 h of exposure to stimulus S6, RPI F vs S6 in this animal showed no significant change (Fig. 5a–d, h–j), but direction selectivity index was significantly increased (Fig. 5d–f, k, l). These results show that exposure to phase-scrambled stimuli in ferrets with some prior visual experience lead to enhancement of smooth-motion direction selectivity, which is consistent with a permissive role of visual experience for the development of direction selectivity.

### Exposure to irregular motion: population analysis

Comparison of RPI and DI before and after visual stimulation for every ferret in the study is shown in Supplementary Fig. 3. In all (4/4) ferrets with no visual experience there was a positive ΔRPI, suggesting increased selectivity for the training sequence following exposure (EO = 0, Supplementary Fig. 3A–D). In 3/4 of these same ferrets, there was a negative or zero ΔDI, suggesting no permissive increase in traditional direction selectivity. Taken together, these results imply that visual experience exerts an instructive role in young ferrets without prior visual experience. In contrast, in the majority (6/8) of young ferrets with several days of visual experience (EO 1–10; Supplementary Fig. 3E–L), ΔRPI was either slightly negative or not different from zero, suggesting lack of selectivity gain in favor of the training sequence. However, in most (7/8) of these slightly more experienced ferrets, there was a significantly positive ΔDI, suggesting increased direction selectivity, just as in animals from our 2008 paper (Supplementary Fig. 3N). Taken together, these results imply that visual experience exerts a permissive role in young ferrets with longer (1–10 days) visual experience. A single older ferret imaged beyond the critical period for direction selectivity development (EO 20; Supplementary Fig. 3M) showed a small decrease in both RPI and DI.

**Fig. 6:**
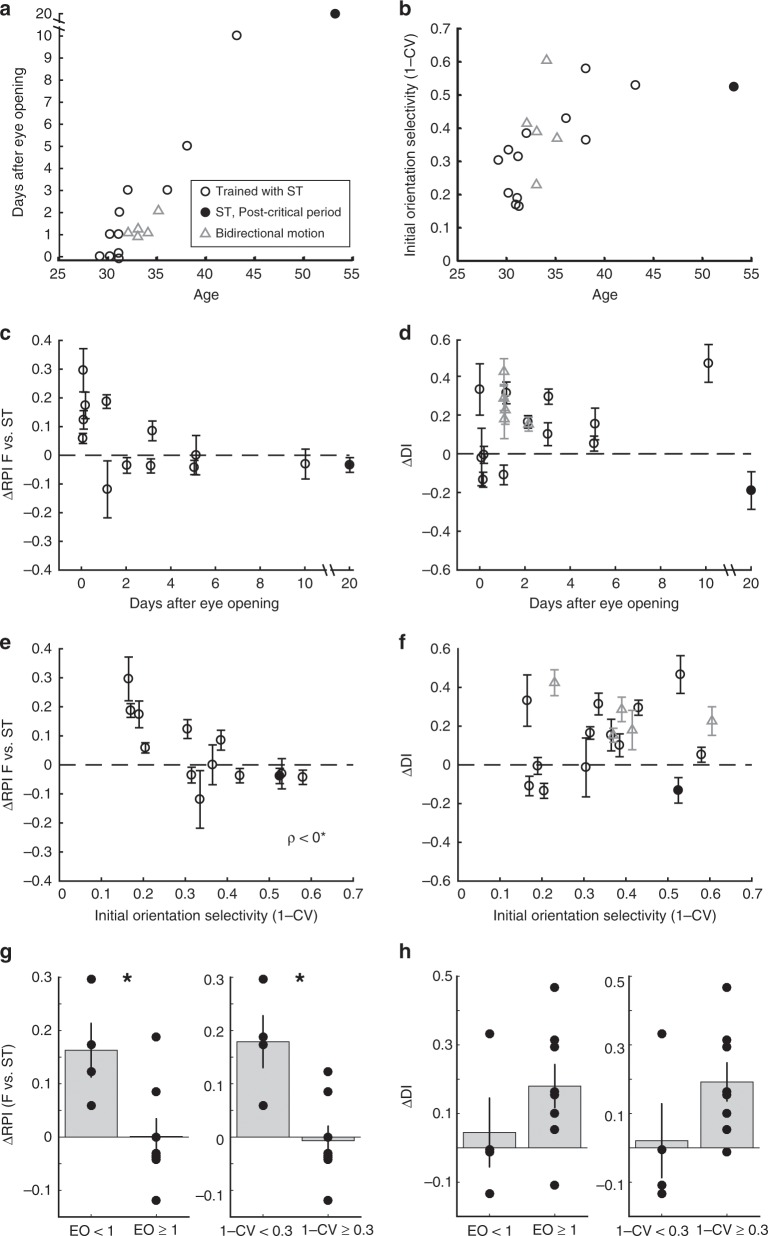
Relationship between changes in visual selectivity and parameters related to animal maturity. **a** Animal age, days after eye-opening. *ST* indicates animals trained with S4, S6. Triangles indicate animals from ref. ^17^ trained with bidirectional moving stimuli. Filled circle is single animal beyond critical period for direction selectivity development. **b** Animal age, initial orientation selectivity (1-CV). On average, orientation selectivity becomes stronger with age, but there is range of initial selectivity in youngest animals, likely reflecting range of cortical maturity achieved. **c** Difference in RPI for F vs trained stimulus (denoted *ST*; S4 or S6) before and after training (error bars 95% confidence intervals) plotted against days after eye-opening (ρ = −0.43, *p* < 0.165, DF = 12-2). **d** Same, but difference in direction index values (*ρ* = 0.54, *p* < 0.068, DF = 12-2). **e** Difference in RPI vs initial orientation selectivity that was measured at beginning of the experiment (before training stimulus exposure). Animals with lowest orientation selectivity exhibit strong changes in RPI, become more selective for scrambled training stimulus. *ρ* < 0* indicates significant negative correlation (*ρ* = −0.71, *p* < 0.009, DF = 12-2). **f** Same, but for DI (*ρ* = 0.44, *p* < 0.152, DF = 12**-**2). **g** Left: Changes in RPI (F vs training stimulus ST) for animals whose eyes were opened by experimenter (EO < 1) and animals whose eyes opened naturally before experiment (EO ≥ 1). * Indicates significant difference, *t*-test (mean ± SEM EO < 1: 0.16 ± 0.05, EO ≥ 1: 0.00 ± 0.03, *p* < 0.020241, DF = 12-2). Right: Changes in RPI (F vs ST) for animals that exhibited low initial orientation selectivity (1-CV < 0.3) and animals that exhibited higher initial orientation selectivity (1-CV ≥ 0.3) * indicates significant difference, *t*-Test (mean ± SEM 1-CV < 0.3: 0.04 ± 0.10, 1-CV ≥ 0.3:: −0.01 ± 0.03, *p* < 0.0045866, DF = 12-2). **h** Same as **g**, but change in direction index values indicated. Left: Difference is not significant (mean ± SEM EO < 1: 0.04 ± 0.10, EO ≥ 1: 0.18 ± 0.06, *T*-test, *p* < 0.25972, DF = 12-2). Right: Difference is not significant (mean ± SEM 1-CV < 0.3: 0.02 ± 0.11, 1-CV ≥ 0.3: 0.19 ± 0.06, *T*-test, *p* < 0.14439, DF = 12-2). Ns are animals (averages across all significantly-responding cells in each animal). The post-critical period animal was excluded in this analysis. RPI exhibited increases in inexperienced animals and in animals with low initial orientation index values.

The above results suggest that the influence of early sensory experience-driven cortical activity on the nascent circuit undergoes a developmental transition—from instructive to permissive—just around the time of natural eye-opening. We wanted to examine which out of a handful of parameters that all reflect different aspects of circuit maturity were correlated with increased selectivity to the phase-scrambled training stimulus or increased direction selectivity. We decided to concentrate on 2 parameters in particular, namely, eye-open status (EO) and initial orientation selectivity. Both these parameters increase with increasing age (Fig. 6a, b), but, because individual ferrets open eyes at different ages and mature at different rates, age alone is not a good predictor for level of experience or circuit maturity. Therefore, we concentrated on correlating ΔRPI and ΔDI with eye-opening status (EO) and initial orientation selectivity. Ferrets whose eyes were opened prematurely (EO = 0) were highly likely (4/4) to exhibit increased selectivity to the phase-scrambled stimulus (Fig. 6c, d), but the hallmark of immaturity that best predicted increased selectivity to the phase-scrambled stimulus was the animal’s initial orientation selectivity index (OSI) value (Fig. 6e). While orientation selectivity is evident at the time of eye-opening, OSI values are relatively small in naive animals and increase substantially over the first 1–2 weeks of visual experience^15,17^. Animals with weak initial OSI values showed large increases in selectivity for the phase-scrambled stimulus and lacked increases in direction selectivity, while animals with stronger initial OSI values (>0.3) generally lacked increases in selectivity for the phase-scrambled stimulus (6/8) and instead exhibited robust increases in direction selectivity (7/8) (Fig. 6e, f). We also analyzed the data by categorizing the ferrets into inexperienced (EO < 1) and experienced (EO ≥ 1) groups, or low (1-CV < 0.3) and high (1-CV ≥ 0.3) initial orientation selectivity groups. The results presented in Fig. 6g, h corroborate that RPI exhibits significantly larger increases in the inexperienced and low orientation selectivity index value groups compared to experienced or high initial orientation selectivity groups.

**Fig. 7:**
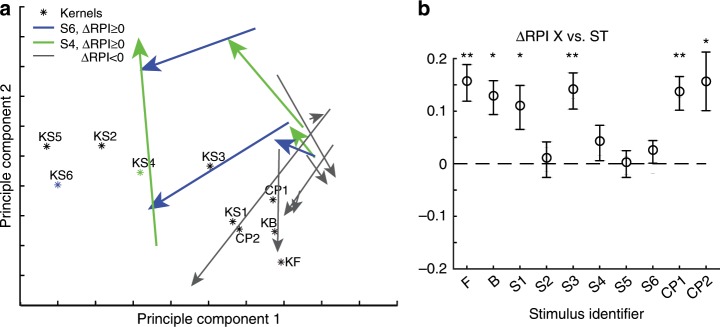
Responses became more similar to those of ideal neurons selective to the training stimulus. **a** Principle component projection from 10-dimensional space to 2-dimensional space of mean responses (for each animal) to the chosen set of 10 stimuli, before and after training, with vectors indicating the transition from the mean response before training to after training (arrow points at mean state after training). Responses of hypothetical neurons optimized for each stimulus (KF, S4, CP2, etc.) shown. Animals that exhibited significant ∆RPI (F vs *ST*) are indicated (trained with S4 green, S6 blue). In this reduced view, average responses of significant animals moved closer to *KST*, while animals (8/8) that exhibited no significant effect moved to be near to KF, KB, KCP1, KCP2 (typical V1 receptive fields). **b** Change in RPI for significant animals with each stimulus used as a reference with the training stimulus (*Sn* vs *ST*). For animals trained with S4 or S6, values of RPI (S4 vs S4) or RPI (S6 vs S6) were excluded from the average as it is 0 by definition. Error bars indicate SEM of the mean. * or ** indicates one-tailed *T*-test (*p < 0.05, **p < 0.005, DF = 6, DF = 3 when X is S4/S6) with mean > 0. Comparison for each RPI (X vs ST) is single comparison evaluating only stimulus X. Changes in responses became more like a hypothetical neuron tuned to the training stimulus *KST* than stimuli F, B, S1, S3, S4, CP1, and CP2, and changes in responses remained equally close to stimuli S2, S5, and S6 (when S6 was not the training stimulus) on average. As responses changed in 10-dimensional space, they moved closer to *KST* for most stimuli while moving no closer or farther from KS2, KS5, and KS6. This is consistent with the idea that the training stimulus provided an instructive influence on receptive field properties in this early developmental period.

While these data showed that the least mature animals acquired receptive fields that were more correlated with the phase-scrambled training stimulus, it remained possible that we were merely pushing the brain circuitry into an unnatural configuration that, while producing increased responses to the phase-scrambled training stimulus, was simply another allowable developmental configuration but not one that was truly instructed by the training stimulus. To understand how responses were altered relative to the full stimulus family, we projected the 10-d responses of these animals onto a reduced 2-d representation using principal component analysis (Fig. 7a). In each case where we observed a significant training effect (full 95% range > 0 in Fig. 6c, e), the mean responses of these animals moved closer to the training stimulus. Further, we performed a pairwise examination of the change in RPI for each stimulus compared to the training stimulus in these animals (Fig. 7b). For most stimuli (F,B,S1,S3,S4,CP1,CP2), the actual responses moved significantly closer to those of a hypothetical neuron that would respond optimally to the training stimulus. For other stimuli (S2, S5, S6 when it was not the training stimulus) that were located near to the training stimuli (S4, S6) in 10-dimensional space (Fig. 7a), the average tuning moved about equally close to hypothetical optimal responses for the reference stimulus and the training stimulus. In total, these results indicated that the neural responses were becoming more like those of hypothetical neurons optimally tuned to the training stimulus, as expected for an instructive influence.

One may be concerned that the enhanced plasticity observed in the animals with least experience and weakest initial orientation index values could be artifactual coincidences if these animals also exhibited poorer quality responses. To address this possibility, we examined signal strength (that is, calcium response strength) and signal-to-noise ratios in Supplementary Fig. 4, and found no correlation of signal strength or signal-to-noise ratio with experience or initial orientation index value. We, therefore, found no evidence that the quality of the responses differed across animals systematically by days of visual experience or initial orientation selectivity.

These results show that the spatiotemporal tuning of neurons was modified by visual experience provided to the premature cortex. However, all response properties were not malleable, indicating that the influence of premature or very early experience has limits. Orientation preference, for example, was not altered by this experience (Supplementary Fig. 5), suggesting that either some features of the circuit are fixed even at our earliest point of examination, or that longer stimulation would be required to alter these properties. Nevertheless, the spatiotemporal response profile of these cells was modified through experience with a stimulus that was specific to the individual animal in a manner that was not possible just a few days later.

## Discussion

In this study, we tested the precise role of visual experience in the development of direction selectivity by exposing young ferrets to phase-scrambled gratings for several hours and asking if the cortical neurons could develop selective responses to such unusual stimuli. We found that in ferrets with no visual experience, V1 neurons developed selective responses to such irregular motion. In contrast, slightly more mature ferrets with 1–10 days of visual experience did not acquire increased selectivity to the phase-scrambled patterns, but instead exhibited a developmentally-typical increase in direction selectivity. We conclude that the influence of visual experience on the developing cortical circuit undergoes a transition—from instructive to permissive—right around the time of natural eye-opening.

To our knowledge, this is the first time that cortical neurons have been induced to become selective to an irregular spatiotemporal stimulus through visual stimulation alone. In the disease amblyopia, a poor alignment of the 2 eyes or poor resolution in 1 of the 2 eyes causes a substantial drop in receptive field acuity/resolution and poor matching of receptive field properties across the 2 eyes^13^, which reflects degradation of receptive field structure but not the formation of a new, precise spatiotemporal receptive field. Other studies have imposed new receptive field structure, but have done so by pairing visual stimuli with external feedback control of visual^31^ or somatosensory^32^ cortex. The neurons in our study exhibited responses unlike those found in typically-developing animals in that they showed specific selectivity for an unnatural, phase-scrambled grating stimulus, which is a strong demonstration of instructive plasticity. This selectivity also differs from the interesting induction of sequence selectivity in visual cortex^33,34^ in that the selectivity introduced here is to a stimulus that is compact in space and time, with a cycle frequency of 4 Hz. The ability of the cortex to acquire such unusual selectivity suggests that the cortex is particularly malleable in the face of activity in this very early window.

It is interesting that there are phenomenological differences among the forms of activity-driven plasticity for spatiotemporal scrambled stimuli (before or at eye-opening), typical direction selectivity (first 2 weeks after eye-opening) and ocular dominance plasticity (excluded until about 2 weeks after eye-opening, then closes about 1 month after eye-opening^35–38^). Functionally, it is useful for ocular dominance plasticity to be excluded around the time of eye-opening, because one of the eyes may open earlier than the other. But spatiotemporal selectivity seems highly plastic immediately before and after eye-opening. These phenomenological differences suggest that these forms of plasticity may be implemented by different mechanisms across development.

While we have shown that visual activity in the window from a few days before eye-opening to just after eye-opening drives strong plasticity in spatiotemporal selectivity, it remains unclear how exactly this plasticity is used by the developing animal under typical developmental conditions. Spontaneous activity, which is necessary for the development of orientation selectivity^9^, dominates in the week before eye-opening^3,12,39^, and it may be the case that the patterns of this spontaneous activity “instruct” the formation of the cortical circuitry that reflects visual tuning parameters such as selectivity to smoothly moving stimuli. This spontaneous activity is sufficient for the formation of orientation selectivity and the initial biases for direction angle preference because both still form in dark-reared animals^15,23^. In addition, very early visual experience through the closed lids drives visual activity^28,29^ and this activity, in addition to experience in the hours after eye-opening, may instruct the development of smooth spatiotemporal receptive fields under typical conditions. Differences in the quality and patterning of activity that typically occurs in this early window may underlie species differences in functional architecture such as ocular dominance patches or receptive field parameters such as the fraction of cells that exhibit direction selectivity^40^.

We conclude that the influence of neural activity on the formation of visual circuits exhibits a sharp transition from instructive to permissive which occurs around the onset of natural visual experience. This conclusion builds on the prior knowledge that spontaneous activity before experience is necessary for proper development of visual circuits^3,9^ by suggesting that the quality and patterning of early activity sculpts the circuitry that supports the parameters of tuning such as spatiotemporal selectivity that are later revealed when selectivity is amplified through experience. After this transition, the net influence of activity-dependent circuit mechanisms must be qualitatively different, because a variety of patterns of neural activity drive the formation of typical smooth direction selectivity, with tuning parameters that cannot be greatly altered.

This developmental transition also mirrors a physiological transition observed in rats and in preterm humans, where flashes of light given before the typical onset of natural visual experience produce prolonged bursts of cortical activity, but these prolonged bursts fade around the onset of natural visual experience (P12 in rats, 36 gestational weeks in humans)^41^. The circuit changes underlying this transition are still unclear, but changes in cortico-thalamic loops and cortical inhibition may contribute^41,42^. Emerging research suggests that preterm humans exhibit higher rates of poor acuity later in life^43^ that cannot be explained by the acuity of the eyes^44^. The mechanisms underlying this poor acuity are not understood and may be varied. Brain damage could contribute^43^. But it is also possible that the premature cortex could be vulnerable to certain types of premature visual experience that could impact the formation of the initial brain circuitry. Future research on the influence of visual stimulation and neural activity on the premature brain may inform best practices for care of the earliest preterm infants.

## Methods

### Animal preparation

All experimental procedures were approved by the Brandeis University’s Institutional Animal Care and Use Committee (IACUC) and performed in compliance with National Institutes of Health guidelines.

### Animal source and housing

Ferrets (*Mustelo putorius furo*) were obtained from Marshall Bio-Resources. Litters of 4 or more kits arrived with a jill at around postnatal day (P) 10. Animals were housed in a room with timed lights (12 h on, 12 h off) in a custom stainless-steel cage (60 cm × 60 cm × 35 cm) with a hammock and small toys. For the entire study, a total of 13 female ferrets were used and all experimental procedures were carried out between postnatal days P19–55. Female ferrets were used because housing mature male ferrets in the same room with mature female ferrets causes stress to the female ferrets.

### Virus injection

GCaMP6s was expressed in ferret V1 by transfection with the virus AAV2/9.Syn.GCaMP6s.WPRE.SV40^30^ obtained from the UPenn Vector Core and later at AddGene (100843-AAV9). A detailed description of virus injection in young ferret kits has been published elsewhere^45^. Briefly, ferret kits (P19-20) were anesthetized with a ketamine-xylazine cocktail and a small craniotomy was made in the left hemisphere over visual cortex following sterilization of the scalp with Betadine and 70% isopropanol while the kit’s head was held in a stereotactic device. Through a very small opening made in the dura, a glass injection micropipette (20–30 μM tip diameter, beveled to ~22° using a Narishige Micropette Grinder EG-400) was inserted into the brain. With a volume injector (Nanoject II, Drummond Scientific), 1 μL of virus solution (~6.3 × 10^12^ genomes/ml) was delivered over two different depths (150 and 500 um from the brain surface). The total volume was injected in small steps of 20–30 nL with 10 to 15 s rests between injections. A 10–14 day recovery period was allowed for GCaMP6s protein to express in visual cortical neurons before imaging experiments were carried out.

### Surgical preparation

The ferret was sedated with ketamine (20 mg kg^−1^ im). Atropine (0.16–0.8 mg kg^−1^ im) and dexamethasone (0.5 mg kg^−1^ im) were administered to reduce bronchial and salivary secretion and to reduce inflammation, respectively. The animal was next anesthetized with a mixture of isoflurane, oxygen, and nitrous oxide through a mask and a tracheostomy was performed. The animal was then ventilated with 1.5–3% isoflurane in a 2:1 mixture of nitrous oxide and oxygen. A cannula was inserted into the intraperitoneal (ip) cavity for delivery of neuromuscular blockers and Ringer solution (3 ml kg^−1^ h^−1^), and the animal was inserted in a custom stereotaxic frame that did not obstruct vision. All wound margins were infused with bupivacaine. Silicone oil was placed on the eyes to prevent corneal damage. Before imaging commenced, the ferret was paralyzed with the neuromuscular blocker gallamine triethiodide (10–30 mg kg^−1^ h^−1^) through the ip cannula to suppress spontaneous eye movements, and the nitrous oxide-oxygen mixture was adjusted to 1:1. The animal’s ECG was continuously monitored to ensure adequate anesthesia, and the percentage of isoflurane was increased if the ECG indicated any distress. Body temperature was maintained at 37 °C.

While the animal’s head was held in the stereotaxic frame, a wire mesh was attached to the skull with VetBond (3 M) glue to provide an anchor point for dental acrylic, and a stainless steel head plate with a 1 cm opening was cemented to the skull above the virus injection site^46^. The head plate was secured to the skull with dental acrylic fortified with VetBond glue and cured with Zip Kicker (ZAP). A small opening in the cranium was drilled through the head plate opening with a dental drill (Medidenta). The dura above the injection site was removed over a 1 mm × 1 mm area for imaging. Warm agarose (3% in 0.1 M phosphate-buffered saline) was poured over the opening and a 5 mm circular coverslip (Warner Instruments) was gently pressed into the solidifying agarose such that the coverslip rim touched the bone around the craniotomy and small amounts of agarose oozed out on top of the coverslip rim to secure it in place.

### Visual stimulation

Visual stimuli were created in MATLAB with the Psychophysics Toolbox^47–49^ on a Macintosh Pro running OS X (10.6, Snow Leopard) and displayed on a Dell monitor 1704FPVt (40 cm viewing distance) and gamma-corrected with a Spyder 3 Express. All stimuli were sinusoidal gratings presented on the full screen at a spatial frequency of 0.8 cycles per degree and a temporal frequency of 2 Hz (discretized in the case of phase-scrambled sequences). All gratings were displayed as 10 repeating cycles for a duration of 5 s, with an interstimulus interval of 5 s. During training, stimuli were displayed 5 s on, 10 s off, in bouts of 20 min, with 10 min of rest (gray screen) after each 20-min bout.

### 2-photon calcium imaging

Cells in V1 were imaged with a 2-photon microscope (Ultima IV, Prairie Technologies, Madison, WI) using 920 nm laser light (Mai Tai Deep See, Spectra Physics) and a ×16 saline-immersed objective (×16, 0.8NA Nikon) with total output power <50 mW. To shield the light of the stimulus monitor from the microscope, the objective and head plate were covered with a custom-sewn sleeve made from light-tight fabric (ThorLabs) and elastic (Michaels Stores Inc.), and augmented as needed with light-tight tape (ThorLabs)^46^. Light block quality was assessed by setting the photomultiplier gain to maximum, shuttering the laser, and scanning while turning the monitor on and off to verify that no modulation could be observed. During visual stimulation, 512 × 512 pixel image frames were acquired continuously every 1.3–1.8 s, and 8 repetitions of each stimulus were recorded.

Cells were imaged in layer 2/3 at depths ranging from 100 to 300 μm. In all cases, we examined the cytoarchitecture at the surface and focused downward to be sure we were lower than cortical layer 1. Before training, images were typically acquired only at a single depth to avoid providing too much experience to the animals during the measurements (each measurement was 20 min). After the training, images were typically acquired at more than 1 depth, and these recordings were displaced in Z by at least 40 μm to avoid recording the same cells twice. All recordings were carefully examined to ensure stability in the Z-axis, and any recordings showing significant Z drift were discarded.

### 2-photon imaging analysis

Images were analyzed with custom software written in Matlab and C. Cells were identified by the experimenter, who selected regions of interest for GCaMP6s with new custom software extensions of our 2-photon analysis tools (http://github.com/VH-Lab/vhlab-TwoPhoton-matlab). The experimenter watched movies of the responses in order to detect neurons that only exhibited strong fluorescence when active, and could select regions-of-interest in any video frame. Small horizontal drift over time was corrected semi-manually with a custom software tool that allowed the user to track and align landmarks in the movies over time (correlation-based methods, which work well for images with stable backgrounds such as those obtained with OGB-Bapta-1 AM^17,50^, did not work well for GCaMP6s). Each frame of every video was examined to ensure excellent drift correction and excellent identification of ROIs, and full manual corrections were allowed by the program. For each frame, the intensity value of each cell was computed by averaging all pixels in a circle of radius ~10 µm that was centered on the soma. These circular regions of interest were smaller than the soma itself to reduce the possible intrusion of signals from the surrounding neuropil. When comparing recording epochs from the same tissue across time, we examined whether it was possible to align the single cell ROIs exactly onto the later recording (our tools are derived from those by SDV in refs. ^17,23^). Sometimes, it was evident that we could do so, and the ROIs were considered to be from the same cells. Any small adjustments or errors were corrected by manual examination and noted by the software tools. Other times, even though the recordings were in very similar X/Y locations, the Z location differed by some 20–60 µm, and it was hard to establish definitively that exact correspondences could be made among ROIs, due in part to the dimness of cells when they were not active. (The experiment in Fig. 3 was a case where we could make several cell correspondences before and after training.) Nevertheless, cells were all recorded at locations that were the same to within a few microns before and after training. We made no attempt to specifically report summary data in cells that were recorded at multiple time points in this paper, and simply examine all cells recorded “before” several hours of stimulus exposure and compare these responses to all cells recorded “after”.

### Software

The Matlab code used to run stimuli and perform analyses are in the Van Hooser lab GitHub distributions (see https://github.com/VH-Lab/vhlab_vhtools/wiki/Installation for installation of all packages).

### Stimulus design

Our goal was to develop a stimulus set that could be delivered at a fixed orientation, in order to drive cortical neurons, but that would exhibit a variety of spatiotemporal patterns within that orientation. We designed sinusoidal gratings that moved in irregular temporal patterns. For this purpose, we discretized one full temporal cycle of a grating into 8 phase steps. In this scheme, one cycle of regular forward or backward motion was created by moving a sine-wave grating following an ordered sequence of 8 phase steps ([1 2 3 4 5 6 7 8] or [8 7 6 5 4 3 2 1]), such that on the 9th step of the temporal sequence the grating arrived at the starting spatial phase. To create irregular motion, the sine-wave grating was moved following a scrambled sequence of 8 steps (e.g., [7 4 8 3 1 6 2 5]) to complete one full cycle. Any such 8-step temporal sequence, regular or scrambled, was repeated 10 times to create a 5 s stimulus. Each phase step was shown for 1/(2 Hz*8) = 0.0625 s to reflect a repetition rate of 2 Hz.

In determining the requisite number of phase steps to define a full grating cycle, we considered 3 factors. First, the duration of each phase step on the screen had to be long enough for the visual system to respond to it. If we used a very high number of phase steps, then the temporal energy of a scrambled phase sequence would be very high and the visual system would not respond. Second, the number of phase steps had to be large enough to generate sufficiently distinct stimuli; if we had chosen 4, for example, then only 6 sequences were possible (including forward and backward). Third, the number also had to be small enough so that the total number of possible combinations was within the practical limits of analysis. We chose 8 phase steps and 2 Hz animation to be a compromise among all of these factors.

A grand total of 40,320 ( = 8!) sequences can be created out of 8 steps with no repeats allowed. However, when a sequence is repeated multiple times, as in showing multiple cycles of the grating, the sequence should be considered in a circular space. Effectively, sequence [1 2 3 4 5 6 7 8] is the same as sequence [2 3 4 5 6 7 8 1]. In the circular space, each sequence will have 8 variants that differ only in the starting phase. Thus, the total number of unique, non-phase-shifted sequences made of [1:8] reduces to 5040 (= 40320/8). Out of these, 2 sequences represent regular motion ([1 2 3 4 5 6 7 8] = forward; [8 7 6 5 4 3 2 1] = reverse), while the rest represent irregular motion.

Before choosing a small number of sequences out of the set of 5040 for experiments, we quantified the degree to which each sequence differed from smooth motion, by calculating 3 separate metrics. First, we calculated the correlation between the sinusoidal phase shifts for all 5040 unique sequences and forward and backward motion (Fig. 1c). The correlation was calculated for all possible phase offsets between the 2 sequences in order to find the value with the highest correlation. For example, sequence [3 2 4 5 6 7 8 1] would be rotated to [1 3 2 4 5 6 7 8] when calculating correlation with [1 2 3 4 5 6 7 8]. Note that the calculation is a sum of products of 2 sinewaves at each phase step (sin(2π((step_1_ − 1)/8)x) * sin(2π((step_2_ − 1)/8)x)), where x is the spatial variable. We calculated phase with x = [0 1 2 … 10]/10. For each sequence, the correlations were calculated against forward and backward smooth sequences separately (Fig. 1c), and then the two values were averaged to obtain an average correlation to smooth motion Supplementary Fig. 1C). Second, we calculated the summed total phase interval for each sequence (Supplementary Fig. 1A, C, D). For sequences representing smoother motion (e.g., [1 2 3 4 5 6 7 8]), the phase intervals or steps between adjacent phases are small, thereby yielding a smaller summed total phase interval value. In contrast, a sequence representing higher degree of irregularity (e.g., [8 3 6 2 7 4 1 5]) would contain larger phase steps and therefore yield a larger summed total phase interval value. Third, we computed the 2-D Fourier spectra for each sequence (Supplementary Fig. 1B) and then calculated the total motion energy content for the sequence by summing the absolute value of all Fourier coefficients excepting the 0 temporal frequency value (static grating).

All 3 measures captured overlapping but slightly different aspects of the irregularity of the sequences. Our goal was to choose a small set of sequences for experiments that covered different levels of irregularity. To do so, we first generated various scatter plots of the 3 irregularity measures against each other (Fig. 1c, Supplementary Fig. 1C, D). From these plots, it is generally evident that sequences with higher summed phase intervals also tended to have lower correlation to smooth motion sequences and higher total motion energy, thereby representing more irregular motion. From these scatter plots, we hand-picked a set of 6 sequences (“scrambled”; S1–6) such that some of them fell on the higher end of dissimilarity to smooth motion and some fell in between. In addition, we included 2 classic counterphase grating stimuli sequences that represent the sum of forward and backward smooth motion (CP1 and CP2), which are not part of the set of 5040 sequences analyzed. We deliberately chose not to include any sequence too similar to smooth motion sequences, because any positive effect of training with such stimuli could simply be the result of smooth motion training.

In Supplementary Fig. 2 we show examples of the 2-D Fourier spectra of some of the chosen 10 sequences. The smooth gratings have energy at a single spatial and temporal frequency (positive and negative), while the scrambled gratings exhibit spatial and temporal energy at several spatial/temporal frequency values and show power at higher temporal frequencies than the smooth versions. This results in these sequences having greater total motion energy.

### Analyses of responses and statistics

The response to each stimulus was calculated as (F_stim_ − F_base_)/F_base_, where F_stim_ is the average response inside the region-of-interest during each frame when the stimulus was on, and F_base_ is the average response during the final 3 s of the interstimulus period before stimulus onset. In the youngest animal, aged P29, responses continued for up to 2 s after the stimulus turned off, and the response was averaged in an interval [0 7] s post-stimulus as opposed to [0 5] s for most experiments.

Cells were categorized as “visually responsive” if an ANOVA test over all stimuli and the blank stimulus yielded *p* < 0.05. Cells/regions-of-interest that were not visually responsive were ignored.

The orientation selectivity index for visually-responsive cells was assessed using 1 minus the circular variance, calculated in orientation space^51^:

$$1 - {\mathrm{{CirVar}}} = \left| {\frac{{\mathop {\sum }\nolimits_k R\left( {\theta _k} \right)exp(2i\theta _k)}}{{\mathop {\sum }\nolimits_k R(\theta _k)}}} \right|$$

where *R*(*θ*_*k*_) is the response to angle *θ*_*k*_ with the response to the blank stimulus subtracted.

Cells were categorized as exhibiting significant orientation tuning if responses to drifting gratings of varying orientations passed Hotelling’s *t*^2^-test with *p* < 0.05^17,52^.

If cells were orientation-selective, then responses were fit with a double gaussian function^52–54^ (Eq. 1):

1 $$R\left( \theta \right) = C + R_pe^{\frac{{ - ang_{diff}\left( {\theta - \theta _{pref}} \right)^2}}{{2\sigma ^2}}} + R_ne^{\frac{{ - ang_{diff}\left( {\theta + 180 - \theta _{pref}} \right)^2}}{{2\sigma ^2}}}$$

where *C* is a constant offset, *θ*_*pref*_ is the preferred orientation, *R*_*p*_ is the above-offset response to the preferred direction, *R*_*n*_ is the above-offset response to the null direction, and $ang_{diff}(x) = min(x,\;x - 360,\;x + 360)$ wraps angular difference values onto the interval 0° to 180°, and σ is a tuning width parameter. The tuning width (half-width at half-height) is equal to $\sqrt {\log 4} \sigma$ (half-width at half height)^53^. For these cells, the direction index value was calculated as DI = (Rp−max(Rn,0))/Rp. The *max* function constrains DI to be at most 1, following^17^.

The selectivity index (SI) for stimulus *n* given responses to stimuli [F, B, S1,…,CP2] was computed as in Eq. 2:

2 $${\mathrm{SI}}\left( {\mathrm{n}} \right) = \frac{{{\mathrm{R}}({\mathrm{S}}_{\mathrm{n}})}}{{{\mathrm{R}}\left( {\mathrm{F}} \right) + {\mathrm{R}}\left( {\mathrm{B}} \right) + {\mathrm{R}}\left( {{\mathrm{S}}1} \right) \ldots {\mathrm{R}}\left( {{\mathrm{S}}6} \right) + {\mathrm{R}}\left( {{\mathrm{CP}}1} \right) + {\mathrm{R}}({\mathrm{CP}}2)}}.$$

The selectivity index could, in principle, vary from 0 (no response to Sn) to 1 (exclusive response to Sn), but in practice it tended to peak at lower values (peaks of about 0.2–0.3) because many of the chosen 10 stimuli are correlated with each other. For example, a cell that responded to forward motion would be very likely to respond to the counterphase stimuli CP1 and CP2, because stimuli CP1 and CP2 are mathematically equal to the sum of gratings drifting past one another: that is, they are mathematically equal to the sum of stimuli F and B.

We developed a second measure that considered the responses to all 10 of the stimuli that we termed the Response Projection Index (RPI). We can imagine neural kernels (KX) that would give a maximal response to each stimulus (X), as shown in Fig. 1e, f, and we can compute the responses of these kernels to each of the 10 stimuli. The kernels were taken to be sinewave X-T profiles that matched the structure of the stimulus, except reversed in time. Responses of a kernel to another stimulus was computed by taking the correlation (with optimal phase alignment) between the stimulus and the kernel, and normalizing the kernel’s response to its preferred stimulus as 1. The RPI describes how close the response of a measured neuron, in the 10-dimensional response space, is to the responses that would be expected from one ideal kernel (KXi) relative to another ideal kernel (KXj). The RPI is therefore a relative measure, and requires 2 referent kernels in addition to the measured responses. For example, a neuron that gives responses that are identical to KF would have an RPI(KF vs KB) value of −1, indicating that the response is close to KF, whereas a neuron that gives responses that are identical to KB would have an RPI(KF vs KB) value of +1. Stimulus S6 is uncorrelated with F and B, and KS6 has an RPI(KF vs KB) value of 0.

Given a 10-dimensional vector of responses **R** of a cell and KX_i_ and KX_j_, we calculated D1 and D2 as in Eq. 3 and Eq. 4:

3 $$D1 = \left\| {R - \left( {R \cdot u_1} \right)u_1} \right\|,\;where\;u_1 = \frac{{R\left( {KS_i} \right)}}{{\left\| {R\left( {KS_i} \right)} \right\|}}$$

and

4 $$D2 = \left\| {R - \left( {R \cdot u_2} \right)u_2} \right\|,\;where\;u_2 = \frac{{R\left( {KS_j} \right)}}{{\left\| {R\left( {KS_j} \right)} \right\|}}$$

where ||x|| is the Euclidean norm of the vector x, and then RPI (KS_i_ vs KS_j_) = (D1 − D2)/(D1 + D2).

All statistics are cited in Table 1.

**Table 1:** Statistics table.

Result	*p*-value	Test employed	Degrees of freedom	Additional information
Non-significant correlation between days of visual experience and ∆RPI (KF vs KST) (Fig. 6c)	*p* < 0.1650	corrcoef	12-2	Post-critical period animal excluded. Empirical correlation coefficient was negative.
Significant correlation between initial orientation selectivity and ∆RPI (KF vs KST) (Fig. 6e)	*p* < 0.009	corrcoef	12-2	Post-critical period animal excluded. Empirical correlation coefficient was negative.
Non-significant correlation between days of visual experience and ∆DI (Fig. 6d)	*p* < 0.0678	corrcoef	12-2	Post-critical period animal excluded. Empirical correlation coefficient was positive.
Non-significant correlation between initial orientation selectivity and ∆DI (Fig. 6f)	*p* < 0.1524	corrcoef	12-2	Post-critical period animal excluded. Empirical correlation coefficient was positive.
Significant difference between inexperienced and experienced animals (Fig. 6G, left)	*p* < 0.020241	ttest2	12-2	
Significant difference between inexperienced and experienced animals (Fig. 6G, right)	*p* < 0.0045866	ttest2	12-2	
Non-significant difference between inexperienced and experienced animals (Fig. 6g, left)	*p* < 0.25972	ttest2	12-2	
Non-significant difference between inexperienced and experienced animals (Fig. 6g, left)	*p* < 0.14439	ttest2	12-2	

### Bootstrap confidence intervals

To examine whether a change in RPI was greater than 0 and to put confidence intervals on this distance, we performed a bootstrap difference analysis for each animal. Assume the population of neurons examined before training was N and the population examined after training was M. We then created 10,000 bootstrap simulations where we drew N neurons from the before population (with replacement), and M neurons from the after population (with replacement), and calculated the difference ∆. We then had a distribution of 10,000 values of ∆. We took the mean of this distribution as the mean difference, and reported the 5% and 95% values of this distribution ∆ as the low and high confidence intervals, respectively. If the low confidence value was greater than 0, then the difference in RPI was said to be significant.

To examine the significance of the correlation between days of experience or initial orientation selectivity index values and ∆RPI or ∆DI, we calculated the *p*-value of the correlation coefficient using the Matlab function corrcoef. *T*-tests in Fig. 6g, h were performed with the Matlab function ttest2.

### Materials

All materials are cited in Table 2.

**Table 2:** Resource table.

Reagent type	Designation	Source or reference	Identifiers	Additional information
Software	Matlab	The MathWorks, Natick, MA	RRID: SCR_001622	
Software	GitHub	GitHub	RRID: SCR_002630	
Software	Psychophysics Toolbox	Psychtoolbox.org	RRID: SCR_002881	
Virus	AAV2/9.Syn.GCaMP6s.WPRE.SV40	UPenn Vector Core (previously) and now AddGene 100843-AAV9	Plasmid is RRID:Addgene_100843	Gift from the GENIE Project & Douglas Kim
Hardware and software	Spyder Express 3	Datacolor		
Microscope	Ultima IV 2-photon microscope	Prairie Technologies (now Bruker)		
Laser	Mai Tai HP Deep See	Spectra-Physics / Newport		
Objective	Nikon 16×	Nikon	CFI75 LWD 16X W	0.8 NA, 3.0 mm WD
Precision injector	Nanoject II	Drummond Scientific		
Objective sleeve fabric	Black nylon, polyurethane-coated fabric	ThorLabs	BK5	
Light-block tape	Black masking tape	ThorLabs	T743–1.0, T743–2.0	
Animals	Ferrets	Marshall Bio-resources	“Conventional” colony	
Beveler	Micropipette grinder	Narishige	EG-400	

### Reporting summary

Further information on research design is available in the Nature Research Reporting Summary linked to this article.

## Supplementary information

Supplementary information

Reporting Summary

Description of Additional Supplementary Files

Supplementary Movie 1

Supplementary Movie 2

Supplementary Movie 3

Supplementary Movie 4

Supplementary Movie 5

Supplementary Movie 6

Supplementary Movie 7

Supplementary Movie 8

Supplementary Movie 9

Supplementary Movie 10

## Data Availability

Raw data are available on the Van Hooser lab website: http://vhlab.org/data
