# Supplementary material for: An early phase of instructive plasticity before the typical onset of sensory experience
Source: Nat Commun. 2020 Jan 2;11:11. doi: 10.1038/s41467-019-13872-1 (PMC6940391; doi:10.1038/s41467-019-13872-1)
Supplement: Supplementary file 1 — Supplementary information [file 41467_2019_13872_MOESM1_ESM.pdf]

An early phase of instructive plasticity in visual cortex before the typical onset of sensory experience.

Roy A, Wang S, Meschede-Krasa B, Breffle J, Van Hooser SD. **Nature Communications**

Supplementary Information

Supplementary Figures

Supplementary Figure 1: Quantitative analysis of phase-scrambled sequences

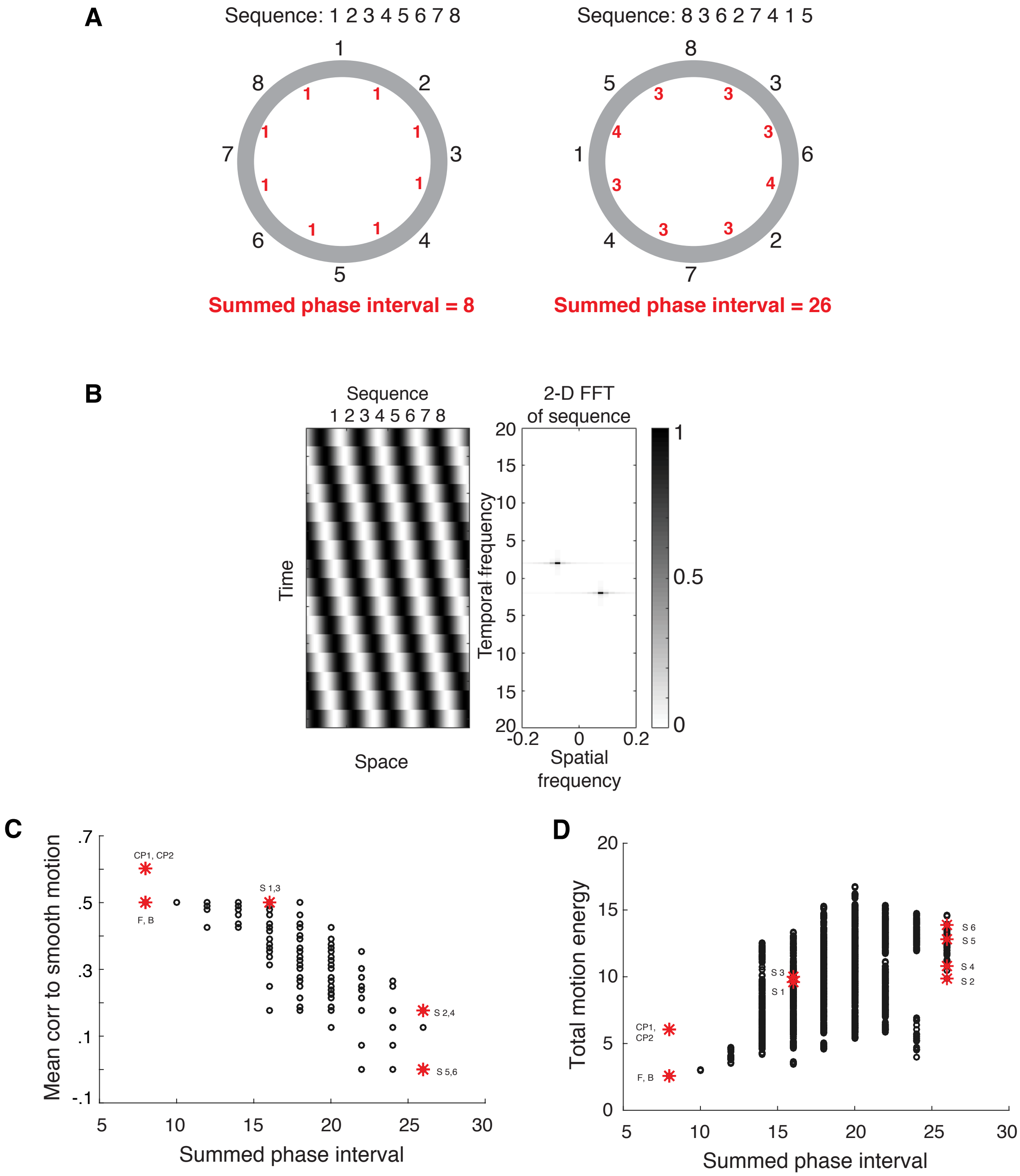

Supplementary Figure 1. Quantitative analysis of phase-scrambled sequences. A): Summed total phase interval. 2 example sequences built out of 8 phase steps are shown in circular space. The phases are shown in black digits, while the absolute value of the differences between two consecutive phases are depicted in red digits. The sum of all the phase intervals are shown below each sequence. **B):** 2-D Fourier spectrum of sequence [1 2 3 4 5 6 7 8]. On the left, 2 cycles of the grating stimulus are shown in space-time, moving following the sequence [1 2 3 4 5 6 7 8]. On the right is shown the corresponding 2-D Fourier spectrum, where each pixel represents the combination of specific spatial and temporal frequencies and the darkness of the pixel represents the normalized Fourier coefficient at those frequency combinations. To calculate total motion energy, all Fourier coefficients within two diagonal quadrants of the 2-D spectrum were summed, except for the pixels corresponding to temporal frequency 0, which represents static grating. **C):** Scatter plot of average correlation to smooth motion against summed phase interval, for all 5040 sequences. The apparent low number of points on the plot is due to very high overlap of these metrics between many sequences. **D):** Scatter plot of total motion energy against summed phase interval, for all 5040 sequences. In both C and D, the locations of the chosen 10 sequences (F, B, S1-6, CP1, CP2) on the space are marked with red asterisks.

Supplementary Figure 2: Spatiotemporal Fourier spectrum of stimuli

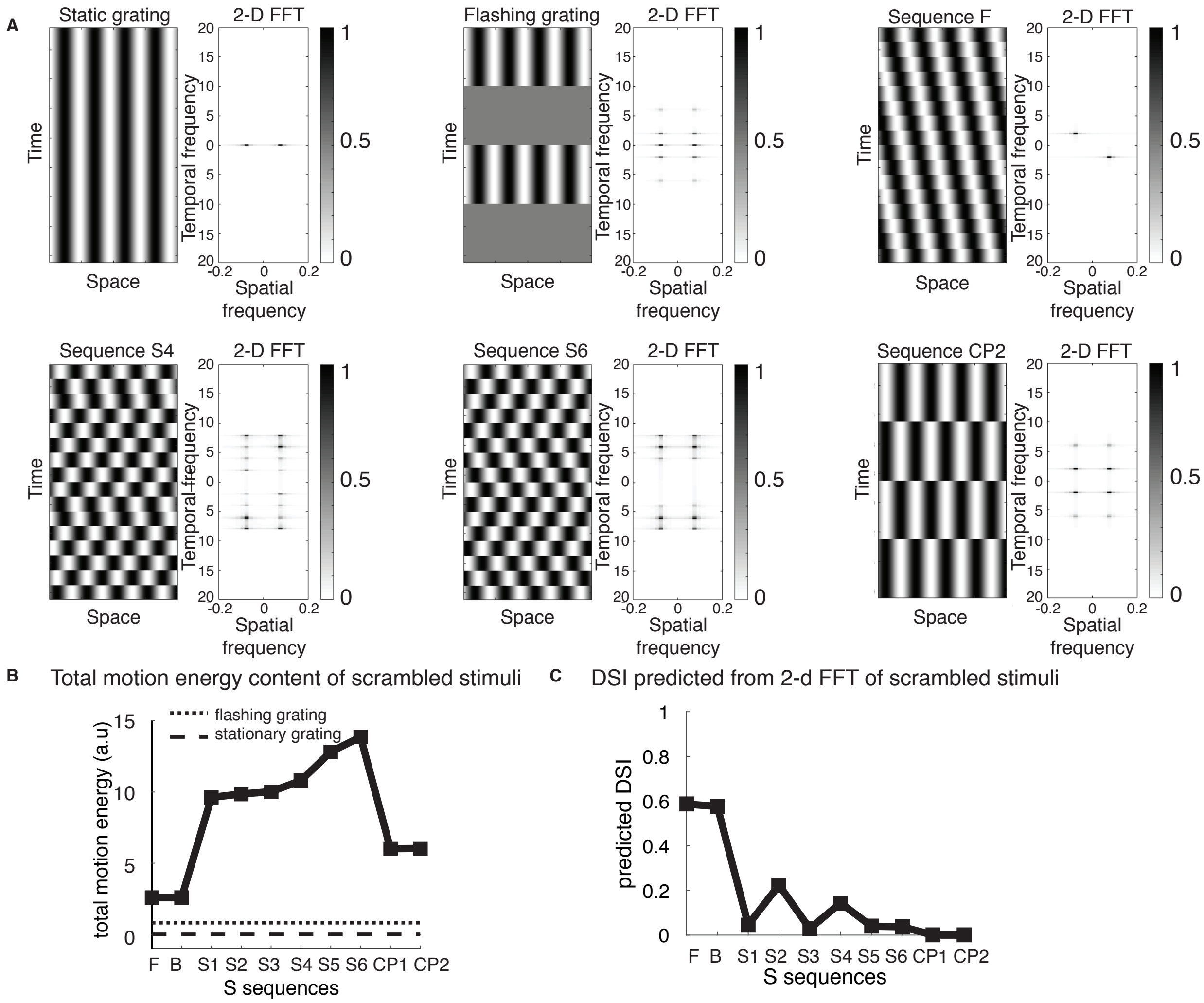

Supplementary Figure 2. Spatiotemporal Fourier spectrum of stimuli. A): 2 cycles of each grating stimulus sequence indicated and the corresponding 2-D Fourier spectrum. A static grating exhibits energy at a single spatial frequency (positive and negative values). A flashing grating exhibits energy at more spatial/temporal frequency values. A smoothly moving grating (F) exhibits energy at a single pair of spatial/temporal frequency values. The phase-scrambled sequences S4 and S6 exhibit energy at a variety of the higher harmonics of the underlying temporal frequency (2Hz), and therefore has energy at more temporal frequencies. **B):** Total motion energy (sum of absolute value of Fourier coefficients excepting the 0 temporal frequency value) of the chosen 10 sequences. **C):** Direction selectivity estimated by convolving each sequence with forward and backward motion and computing (Response_Pref – Response_Opposite) / Response_Pref.

Supplementary Figure 3: Changes in stimulus selectivity for all animals in the study

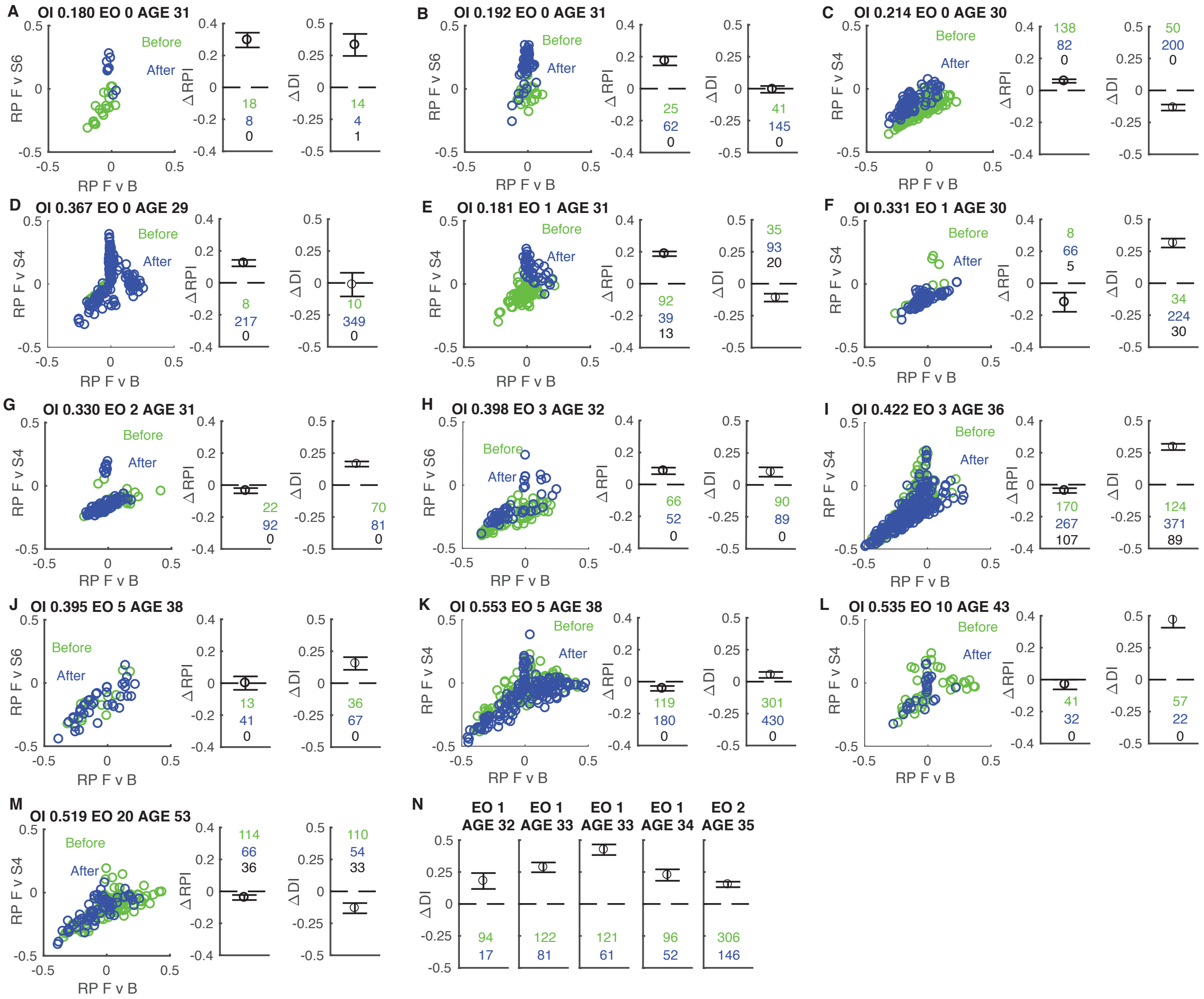

Supplementary Figure 3. **Changes in stimulus selectivity for all animals in the study.** Animals are ordered by days of visual experience (EO) and initial orientation index. **A-M**) Left: Response Projection Index (RPI) for F vs B (X axis) and F vs the trained stimulus (S4 or S6, depending) (Y axis) for cells measured before (green) and after (blue) 6 hours of experience with the training stimulus. Days after eye opening (EO) are noted, as is the animal's postnatal age in days. Middle: Estimated difference in RPI (F vs. trained stimulus) before and after experience (95% confidence intervals). Cell N before (green) after (blue) and number of cells tracked across the imaging sessions (black) indicated. Right: Estimated difference in DI (F vs. trained stimulus) before and after experience (95% confidence intervals). Cell N before (green) after (blue) and number of cells tracked across the imaging sessions (black) indicated. We noted that, on average, animals with fewer days of natural visual experience were more likely to exhibit increased selectivity to an arbitrary stimulus that was presented for 6 hours, whereas animals with several days of visual experience were less likely to exhibit increased selectivity to this stimulus. Instead, animals that had 1-10 days of visual experience often exhibited increases in basic direction selectivity rather than selectivity for the phase-scrambled stimulus. **N)** Change in

direction selectivity in 5 experiments from Li/Van Hooser et al. (2008). All animals exhibited robust increases in direction selectivity when bidirectional moving stimuli (3-6 hours) were used as the training stimulus.

Supplementary Figure 4: Lack of significant correlation of days of visual experience or initial orientation selectivity with signal-to-noise or signal strength

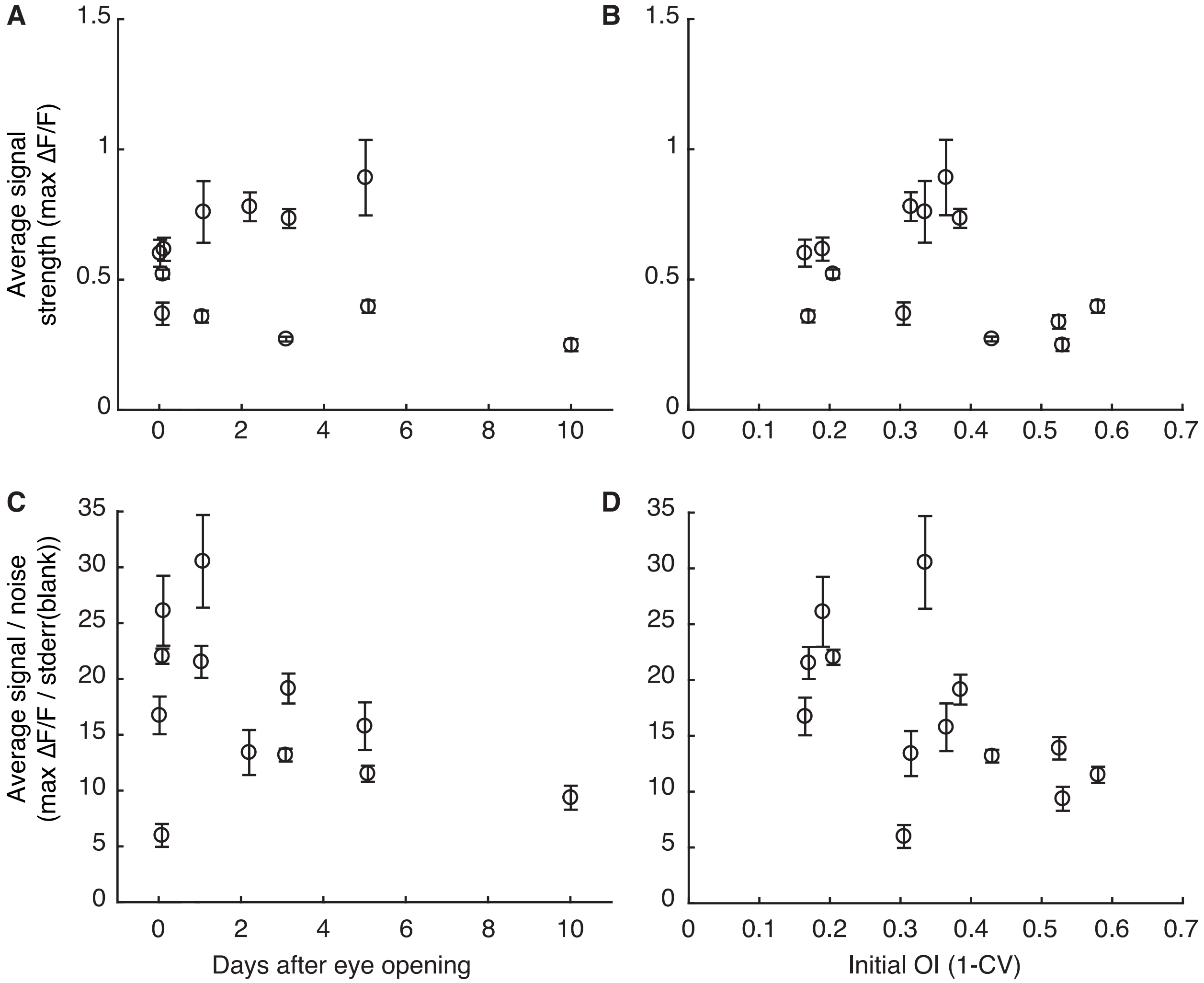

Supplementary Figure 4. **Lack of significant correlation of days of visual experience or initial orientation selectivity with signal-to-noise or signal strength.** Initial average signal strength (mean ΔF/F response to the preferred stimulus after blank stimulus response subtraction) plotted against **A)** days after eye opening (Correlation test: p=0.4516, DF = 12-2) and **B)** initial orientation index (Correlation test: P=0.4081, DF = 12-2). Signal to noise ratio defined as signal strength (defined as in **A** and **B**) divided by the standard error of the mean of the response to the blank stimulus plotted against **C)** days after eye opening (Correlation test: P=0.1334, DF = 12-2) and **D)** against initial orientation index (Correlation test: P=0.0939). The average value of these quantities was computed across all cells for each animal, and the mean and standard error of the mean are shown as each data point on the graph. These results indicate that the results of the study cannot reflect very poor or noisy responses in the youngest or most immature animals.

Supplementary Figure 5: No changes in orientation preferences over training

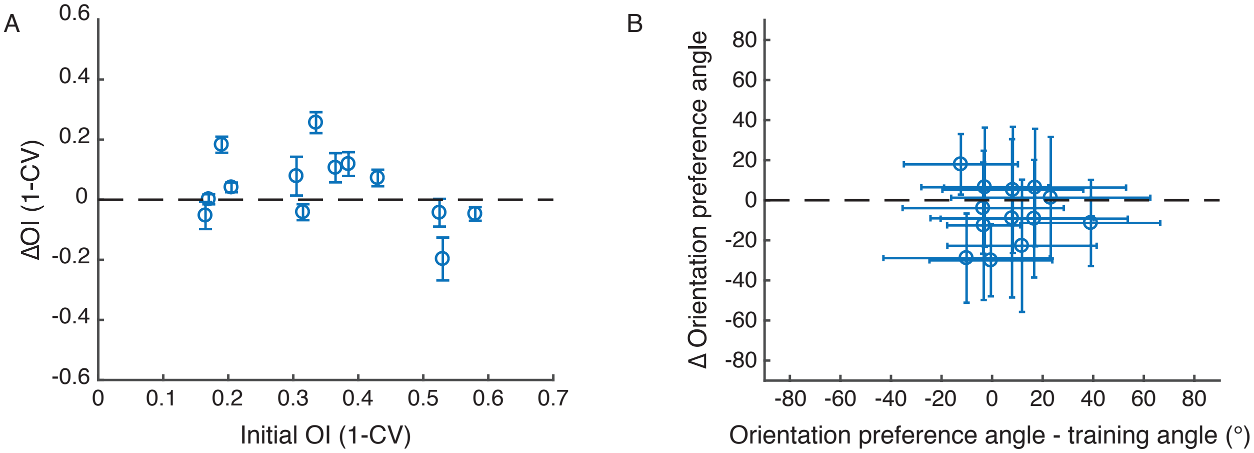

Supplementary Figure 5. **No changes in orientation preferences over training. A)** Changes in orientation selectivity index values before and after training as a function of initial orientation selectivity. On average, orientation selectivity index values increased slightly after training, but not consistently from case to case. Mean and bootstrap standard error is shown for each animal. **B)** Changes in orientation preference values for each animal with respect to initial orientation preference angle, with circular standard deviation shown (in degrees). The orientation preference angle was rotated so that the training orientation was 0. If a strong change in orientation preference were evident, the line should have a negative slope (so that negative rotated orientations would be increased, and positive rotated orientations would be decreased). Instead, there is no correlation. Correlation test: P=0.987, degrees of freedom = 13-2.
