# Supplementary material for: An early phase of instructive plasticity before the typical onset of sensory experience
Source: Nat Commun. 2020 Jan 2;11:11. doi: 10.1038/s41467-019-13872-1 (PMC6940391; doi:10.1038/s41467-019-13872-1)
Supplement: Supplementary file 2 — Reporting Summary [file 41467_2019_13872_MOESM2_ESM.pdf]

nature research

Corresponding author(s): Stephen D. Van Hooser

Last updated by author(s): Nov 28, 2019

Reporting Summary

Nature Research wishes to improve the reproducibility of the work that we publish. This form provides structure for consistency and transparency in reporting. For further information on Nature Research policies, see Authors & Referees and the Editorial Policy Checklist.

Statistics

For all statistical analyses, confirm that the following items are present in the figure legend, table legend, main text, or Methods section.

n/a	Confirmed	
☐	☒	The exact sample size (*n*) for each experimental group/condition, given as a discrete number and unit of measurement
☐	☒	A statement on whether measurements were taken from distinct samples or whether the same sample was measured repeatedly
☐	☒	The statistical test(s) used AND whether they are one- or two-sided<br>*Only common tests should be described solely by name; describe more complex techniques in the Methods section.*
☐	☒	A description of all covariates tested
☐	☒	A description of any assumptions or corrections, such as tests of normality and adjustment for multiple comparisons
☐	☒	A full description of the statistical parameters including central tendency (e.g. means) or other basic estimates (e.g. regression coefficient) AND variation (e.g. standard deviation) or associated estimates of uncertainty (e.g. confidence intervals)
☐	☒	For null hypothesis testing, the test statistic (e.g. *F*, *t*, *r*) with confidence intervals, effect sizes, degrees of freedom and *P* value noted<br>*Give P values as exact values whenever suitable.*
☒	☐	For Bayesian analysis, information on the choice of priors and Markov chain Monte Carlo settings
☒	☐	For hierarchical and complex designs, identification of the appropriate level for tests and full reporting of outcomes
☐	☒	Estimates of effect sizes (e.g. Cohen's *d*, Pearson's *r*), indicating how they were calculated

Our web collection on statistics for biologists contains articles on many of the points above.

Software and code

Policy information about availability of computer code

Data collection	Matlab, Psychtoolbox, PrairieView, and our custom code at http://github.com/VH-Lab
Data analysis	Matlab and our custom code at http://github.com/VH-Lab

For manuscripts utilizing custom algorithms or software that are central to the research but not yet described in published literature, software must be made available to editors/reviewers. We strongly encourage code deposition in a community repository (e.g. GitHub). See the Nature Research guidelines for submitting code & software for further information.

Data

Policy information about availability of data

All manuscripts must include a data availability statement. This statement should provide the following information, where applicable:

- Accession codes, unique identifiers, or web links for publicly available datasets
- A list of figures that have associated raw data
- A description of any restrictions on data availability

All data are available at http://vhlab.org/data

Field-specific reporting

Please select the one below that is the best fit for your research. If you are not sure, read the appropriate sections before making your selection.

☒ Life sciences ☐ Behavioural & social sciences ☐ Ecological, evolutionary & environmental sciences

For a reference copy of the document with all sections, see nature.com/documents/nr-reporting-summary-flat.pdf

Life sciences study design

All studies must disclose on these points even when the disclosure is negative.

Sample size	Our sample size of 13 was larger than initially expected. In previous studies (Li/VanHooser et al. 2008), 5 animals were sufficient to see a large effect. But we saw a surprising effect, and then doubled our sample size to ensure the reliability of the results.
Data exclusions	No exclusion
Replication	After 7 experiments had been performed, we surmised the effect. Then 6 additional experiments were carried out. These experiments were consistent with the initial 7.
Randomization	Experiments were planned on a given day; ferrets had either opened their eyes or not (there is variation in eye opening). This natural variation as well as the variation in the day of birth and the availability of the microscope contributed the random variation.
Blinding	The division of animals into "immature" and "mature" was not done until after the fact (that is, the dependency was a surprise), so investigators were "blind" to the meaning of this condition.

Reporting for specific materials, systems and methods

We require information from authors about some types of materials, experimental systems and methods used in many studies. Here, indicate whether each material, system or method listed is relevant to your study. If you are not sure if a list item applies to your research, read the appropriate section before selecting a response.

Materials & experimental systems

n/a	Involved in the study	
☒	☐	Antibodies
☒	☐	Eukaryotic cell lines
☒	☐	Palaeontology
☐	☒	Animals and other organisms
☒	☐	Human research participants
☒	☐	Clinical data

Methods

n/a	Involved in the study	
☒	☐	ChIP-seq
☒	☐	Flow cytometry
☒	☐	MRI-based neuroimaging

Animals and other organisms

Policy information about studies involving animals; ARRIVE guidelines recommended for reporting animal research

Laboratory animals	Species: Ferret (Mustelo putorius furo). Sex: female. Age: Postnatal day 29-55. N = 13.
Wild animals	N/A
Field-collected samples	N/A
Ethics oversight	Brandeis University Institutional Animal Care and Use Committee (IACUC)

Note that full information on the approval of the study protocol must also be provided in the manuscript.
