# Supplementary material for: An early phase of instructive plasticity before the typical onset of sensory experience
Source: Nat Commun. 2020 Jan 2;11:11. doi: 10.1038/s41467-019-13872-1 (PMC6940391; doi:10.1038/s41467-019-13872-1)
Supplement: Supplementary file 3 — Description of Additional Supplementary Files [file 41467_2019_13872_MOESM3_ESM.docx]

**Description of Additional Supplementary Files**

File name: Supplementary Movie 1
Description: Stimulus F

File name: Supplementary Movie 2
Description: Stimulus B

File name: Supplementary Movie 3
Description: Stimulus S1

File name: Supplementary Movie 4
Description: Stimulus S2

File name: Supplementary Movie 5
Description: Stimulus S3

File name: Supplementary Movie 6
Description: Stimulus S4

File name: Supplementary Movie 7
Description: Stimulus S5

File name: Supplementary Movie 8
Description: Stimulus S6

File name: Supplementary Movie 9
Description: Stimulus CP1

File name: Supplementary Movie 10
Description: Stimulus CP2
